# Efficient Addition of Waste Glass in MK-Based Geopolymers: Microstructure, Antibacterial and Cytotoxicity Investigation

**DOI:** 10.3390/polym13091493

**Published:** 2021-05-06

**Authors:** Giovanni Dal Poggetto, Michelina Catauro, Giuseppina Crescente, Cristina Leonelli

**Affiliations:** 1 Department of Engineering “Enzo Ferrari”, University of Modena and Reggio Emilia, Via P. Vivarelli 10, 41125 Modena, Italy; cristina.leonelli@unimore.it; 2 Department of Industrial Engineering, University of Campania “Luigi Vanvitelli”, Via Roma 29, 81031 Aversa, Italy; michelina.catauro@unicampania.it; 3 Department of Environmental, Biological and Pharmaceutical Sciences and Technologies, University of Campania “Luigi Vanvitelli”, via Vivaldi 43, 81100 Caserta, Italy; giuseppina.crescente@unicampania.it

**Keywords:** waste glass, alkali activation, metakaolin, antibacterial properties, cytotoxic effects, microstructure

## Abstract

Reuse of waste glass can significantly decrease the quantity of waste to be treated or disposed of in landfills, allowing to both diminish the ecological damage and to reduce the costs of transportation for removal. Geopolymer mixes with diverse percentages (20, 50 and 60 wt%) and with different grain size ranges (37 μm < diam < 53 μm; 75 μm < diam < 105 μm) of waste glass and the residual part of pure metakaolin were prepared by addition of NaOH and sodium silicate as alkaline activator solutions. The effect of waste glass on the mechanical and microstructure of new geopolymers has been explored in this study. Fourier transform infrared spectroscopy (FTIR) evidenced the reactivity of waste glass in terms of Si–O and Si–O–Al bonds, more evident for the finer waste glass powder. The consolidation of the materials has been established by reduced weight loss in water and decreased pH and ionic conductivity of the eluate after 7, 14 and 28 days of curing at room temperature. The decrease of the mechanical properties with waste glass content was less evident for the finer glassy powders, yet the value of about 4-5 MPa indicates their potential use as non-structural materials. The consolidated final materials were tested for their effects on the microbial growth of *Escherichia coli* and *Enterococcus faecalis* after 24 and 48 h, respectively. The samples showed a very limited and absent inhibition zone, for fine and coarse grain size ranges, respectively. Finally, the cytotoxicity tests accomplished the ecological valuation of the final consolidated products.

## 1. Introduction

The recycling behavior (e.g., separating glass, plastic, and paper) of EU citizens is strongly increasing. Additionally, container glass manufacturers have been promoting the recycling of this type of packaging material over the years, reaching the record rate of 76% in 2017 (as averaged for 28 member states of the European Union (EU)), as announced in October 2019 by the European Container Glass Federation (FEVE) [1]. Being conscious that glass recovering allows the container glass industry to strongly decrease its ecological footmark by redeemable energy and raw materials, FEVE aims to achieve a 90% collection rate for glass in the European Union by 2030 [2,3]. The 160 glass plants located in the EU deliver more than 50% of their products within 300 km, and more than 70% of raw materials travel less than 300 km.

Producing glass packaging for the food and beverage sector, in addition to pharmaceuticals and cosmetics with recyclable and reusable materials favors lower natural resource consumption, waste, and energy usage in agreement with the United Nations’ Sustainable Consumption and Production Goal-SDG 12 [4]. Nevertheless, glass-recycling directly lets the industry intensely decrease energy consumption and CO_2_ emissions in line with the Climate Action Goal-SDG 13 [5]. Good quality cullet production is industrially achieved through the optimization of separation and purification technologies. Italian data (elaborated from: CoReVe Piano Specifico di Prevenzione 2020) [6] indicates that the waste amount over the recycled amount decreased from 13.72% to 9.93% from 2018 to 2019. Still, more than 220 k tons are diverted to other industries (sanitary and ceramic tiles) during the recycling procedure or even disposed of. The amount usually disposed of is typically the fine dust collected from the many filters positioned all around the recycling plant.

CoReVe [6] (the Italian consortium of container glass producers) reports the use in construction (or in other sectors) of glass not suitable for recycling in glass smelters as an alternative to landfilling. The recovery and treatment process, essential for glass packaging waste transformation into secondary raw material (end-of-waste glass), as it is known [7], determines a parallel production of waste glass, characterized by a high presence of “fine” glass powder (generally <6–10 mm), which is separated from the coarse fraction recycled in the smelter. It should be emphasized that the percentage of “fine” material has been steadily increasing over the years essentially for two reasons:

(i) The recent increase of the “door to door” home collection method, which involves greater fragmentation of the glass;
(ii) Excessive material handling typical of “multi-material” collections, which requires the “pre-selection” phase, after the collection, preparatory to the treatment/valorization of the glass fraction alone.

Many papers dealt with the use of these fine glass powders in several formulations of building materials [8,9,10,11], and we can generalize these studies indicating the following:

(i) The waste fraction below approx. 1 mm is used “as it is” (i.e., without any preliminary cleaning operation) in construction [12];
(ii) The coarser fraction is used in construction with an adequate elimination of the “light” polluting components, such as paper or plastic deriving from the bottle labels [11,13].

On the other hand, we are assisting an increasing interest in room consolidated materials obtained by the alkali activation of aluminosilicate powders, also known as geopolymers, which are suitable for the incorporation of quite a lot of various wastes produced from different sources [14,15,16], among which wastes of glasses is just one. As reported by the review paper by Luhar et al. [17], the process of geopolymerization is capable of producing various inorganic binders containing waste glass which are suitable for different applications (mortars, pre-cast, etc.) in the field of sustainable infrastructures and construction industries. Thus, when geopolymers are opportunely designed and formulated employing waste glass as aggregates or as a silicate source for accessible alkaline activators, a durable, ceramic-like, and sustainable as well as cost-effective building material can be proposed [18].

In the present study, differing from what was previously proposed [8,19], we investigated the effect of untreated waste glass grain size on the alkali activated materials obtained using metakaolin as binder at room temperature. During room temperature curing, container glass waste is not considered reactive because the soda-lime silicate, with SiO_2_ as major component of container glass being very difficult to dissolve in the common media at low temperatures [20]. Thus, only the finer grain size could act as source of silica in substitution to or in addition to the waterglass activator, as indicated by study of Torres-Carrasco et al. [21,22] whilst the coarser fraction could behave as substitute for sand, as inert aggregate [23]. Our innovative approach proposes only the grinding operation on the as-received waste glass from containers separate collection, avoiding the two common pre-treatments of washing and drying. We adopted two different grain sizes, one fine (37 µm < diameter of waste glass particles, d_WG_ < 53 µm) and one coarse (75 µm < d_WG_ < 105 µm) in order to evaluate when the glass particles will finally take part in the dissolution reaction due to their propinquity with the alkaline solution in the matrix. Our main aim was to reach a clear understanding of the differences between the geopolymer gel with reactive glass and with glass as aggregates.

To retain a low energy-consuming process, we cured our geopolymerized solid samples at room temperature, even though several authors propose a moderate temperature curing for waste glass containing geopolymer, i.e., 40 °C [24], 60 °C [25], 80 °C [26], 90 °C [27], 100 °C [28]. It has been assumed that the mechanical performance of the final products has not been optimized, but we intended to favor the sustainability approach.

Having in mind the most sustainable process, the amount of waste glass that we added to the geopolymer mix was in the range 20–60 wt%, starting from the threshold of 20% used in previous studies [29] and trying to increase the maximum level of 50% proposed by Zhang et al. 2020 [28]. Different from the work of Labrincha’s research group [30], we also accurately tested the antimicrobial activity and the cytotoxicity of the final consolidated material to assess its safe use.

## 2. Materials and Methods

### 2.1. Materials

The metakaolin (MK) used in this study was a worldwide used one for geopolymer preparation, i.e., Argical™-M 1000 by Imerys (Paris, France), produced in a rotary kiln (cement type kiln) and sold in form of a powder after grinding of about d_50_ 10–15 µm (particle size cumulative curve is presented in Supplementary Materials as Figure S1). Mineralogical composition [31,32] indicates the presence of traces of anatase TiO_2_, illite (K,H_3_O)(Al,Mg,Fe)_2_(Si,Al)_4_O_10_[(OH)_2_,(H_2_O)], and α-quartz SiO_2_ in a particularly amorphous material with chemical composition: SiO_2_ 53.67; Al_2_O_3_ 39.88; K_2_O 0.68; Fe_2_O_3_ 1.45, TiO_2_ 2.22 (MgO+CaO+Na_2_O+P_2_O_5_) 0.66 (wt%), LOI 1.22% (wt%) as expected in an almost pure metakaolin [32].

Waste glass (WG) powder was obtained from dry grinding of as-received container glass. Its chemical composition was determined by means of Energy Dispersive X-Ray Fluorescence (EDXRF) spectroscopy using a Shimadzu Spectrometer EDX-720 (GmbH, Duisburg, Germany) equipped with 50 W Rh target x-ray tube, a high-energy resolution Si (Li) detector, and five primary x-ray filters. Such composition resulted to be: SiO_2_ 69.29; Al_2_O_3_ 1.93; K_2_O 0.91; Fe_2_O_3_ 0.54; CaO 13.03; Na_2_O 12.15; MgO 1.60 (wt%) and L.O.I. (Loss of Ignition) 0.55 (wt%). The contaminants present in the as-received waste glass are very limited (about 0.5-0.6 wt%) and were characterized by leaching tests according to current regulation: leaching in deionized water, acetic acid 0.5 M (to simulate acidic rains); NaCl solution 3,5% (simulating seawater) according to UNI EN 12457-2 [33], UNI EN 10802:2013 [34], EPA 6020A:2007 [35]. Heavy metals leaching from these three tests indicated only the presence of iron and traces of zinc, deriving from the caps. The organic fraction was characterized according to EPA 3535A - SOLID-PHASE EXTRACTION (SPE) [36] to be composed of acrylic and phthalic acids typical of the adhesive substances used to glue labels to glass bottles.

The two final waste glass powders adopted for this study were sieved in order to obtain two different grain size ranges, the fine one: 37 µm < d_WG_ < 53 µm, and the coarse one: 75 µm < d_WG_ < 105 µm (diameter of the waste glass powder: d_WG_). The grain size for both powders was confirmed by Mastersizer 2000 (Malvern Instruments Ltd., Malvern, UK) and cumulative volume is described in the Supplementary Materials as Figure S1, where also the powder of the as-received metakaolin is presented.

Sodium hydroxide (NaOH), sodium silicate (Na_2_SiO_3_) with SiO_2_/Na_2_O = 2.58, acetone (C_3_H_6_O), and KBr of analytical grade were purchased from Sigma-Aldrich (St. Louis, MO, USA). MilliQ water was used for NaOH 8 M preparation and the sample analyses. The choice of NaOH 8 M solution was decided on the basis of the mechanical performance of MK-based geopolymers [37], since the concentration of 6 moles per liter gave lower values [32].

### 2.2. Preparation of Geopolymer Specimens

The formulation of the geopolymer based on metakaolin, sodium silicate and NaOH was optimized in a previous paper [37]. To the amount of 100 g of MK, dry powder, the addition of 48 mL of NaOH plus 68 mL of sodium silicate was performed under mechanical stirring to obtain the reference geopolymer, GP. In this formulation, the MK powder was substituted by 20, 50, and 60 wt% of ground waste glass, with the two different grain sizes (37 µm < d_WG_ < 53 µm and 75 µm< d_WG_ < 105 µm), to produce the geopolymer composites of the two GP/WG series with fine and coarse particle sizes (GP/WG20%, GP/WG50%, GP/WG60%). The fresh paste was poured into plastic tubes with cap, reaching a height that is twice the diameter of the base (20 mm in diameter and 40 mm in height). After removing all the bubbles, the molds were carefully closed and geopolymers cured at room temperature at 100%relative humidity. The plastic molds were opened after 7 days (or 14 or 28 days) of curing time to proceed with the proper characterization. A minimum of 10 samples per each formulation were obtained.

### 2.3. Geopolymers Characterization

#### 2.3.1. Integrity Test

MilliQ water (1:100 solid–water weight ratio) was added to sample, amounts ranging from 1.51 to 2.34 g. After 24h, the water was removed and the integrity was evaluated estimating: (i) final pH of the eluate; (ii) the samples’ smoothness; (iii) the samples’ hardness and finger pressure; (iv) eluate transparency; (v) weight loss after immersion, adopting the acetone drying procedure to avoid thermal treatments [38].

#### 2.3.2. pH and Ionic Conductivity Measurements

Ionic conductivity and pH measurements were performed with (Crison GLP31 and Crison GLP 21, respectively, HACH LANGE SPAIN, S.L.U, Barcelona, Spain). MilliQ water (1:10 solid–water ratio) was added to the ground and sieved geopolymer samples with both WG grain sizes. After shaking the solution, a time was allowed to elapse in order to sediment the solids prior to analyses. pH and ionic conductivity were collected at different times over the first 48 h: t_1_ = 0 h, t_2_ = 5 min, t_3_ = 10 min, t_4_ = 20 min, t_5_ = 2 h, t_6_ = 4 h, t_7_ = 6 h, t_8_ = 24 h, and t_9_ = 48 h, respectively.

#### 2.3.3. FT-IR Analysis

FT-IR analysis was performed in the range of 400-4000 cm^−1^ by means of the spectrometer Prestige-21, Shimadzu Europe (GmbH, Duisburg, Germany), furnished with a DTGS KBr (deuterated tryglycine sulfate with potassium bromide windows) detector, resolution of 2 cm^−1^ (45 scans). The analysis procedure adopted KBr disks (198 mg of KBr and 2 mg of consolidated geopolymer, or as-received WG and MK). The analyses were executed on the samples extracted after 7, 14, and 28 days at room temperature. FT-IR spectra were elaborated by IR solution and Origin 8 software.

#### 2.3.4. Mineralogical Composition

Crystalline phases of the geopolymers and as-received raw materials (MK and WG) were identified from X-ray diffraction (XRD) attained with a X’Pert PRO, PANAlytical, (Malvern Panalytical Ltd., Malvern, UK) diffractometer operated at 40 kV and 40 mA using Cu-Kα radiation (Ni filtered). Diffraction patterns were collected by the X’Celerator detector from 5 to 70° 2θ with a step size of 0.02° 2θ and a counting time of 3 s. Mineral phases were identified by comparing the experimental peaks with reference patterns (DIFFRAC plus EVA software, 2005 PDF2, Bruker, Billerica, MA, USA).

#### 2.3.5. Microstructural Observation

Fracture surfaces of the hardened geopolymers were studied with ESEM Quanta-200 (FEI Company-Oxford Instruments, Hillsboro, OR, USA) scanning electron microscope with 25 kV accelerating voltage, 3.5 μA emission current, and 20 nA beam current. Portions of the fractured geopolymer sections were coated by gold (10 nm of thickness), using a Gold Sputter Coater, Emitech K550 (London, UK), and placed on an Al stub with conductive carbon glue. The chemical composition of the geopolymer gel was investigated using an Energy Dispersive Spectroscopy (EDS) Analysis X-EDS Oxford INCA-350 (Oxford Instruments, Austin, TX, USA). For semiquantitative evaluation, each specimen’s surface was examined in different spots and averaged, with the confidence interval found to be ±1 wt%.

#### 2.3.6. Mechanical Properties

To test the mechanical properties of the waste glass with added MK-based geopolymers, compression tests were performed with an Instron 5567 Universal Testing Machine (Norwood, MA, USA) after 28 days of curing. For the tests, cylindrical samples with a diameter of 20 mm and a height of 40 mm were used. The load (30 kN load limit) was applied and increased by displacement rate of 1 mm/min. The tests were executed in displacement control mode at a constant loading velocity and no preload. They were stopped after obtaining 3 valid tests for each different geopolymer composition. Compressive strength values are assumed to be the mean value of three tests attended with the 2% variance.

#### 2.3.7. Antibacterial Activity

In order to estimate the antibacterial properties of the geopolymers, *Escherichia coli,* (ATCC 25922) from Gram-negative family, and *Enterococcus faecalis,* (ATCC 29212) from Gram-positive family, were grown in the absence and presence of the synthesized materials extracted after 7, 14 and 28 curing days at room temperature. Samples used for the analyses were finely ground and pressed to obtain disks of 100 and 200 mg of weight that were radiated by UV light for 1 h for sterilization. The bacterial suspension of 10^5^ CFU/mL was obtained by diluting the strains in distilled saline water (0.9% NaCl). After plating *E. coli* in TBX Medium (Tryptone Bile X-Gluc) (Liofilchem, Italy) and *E. faecalis* in Slanetz Bartley agar base (Liofilchem, Italy), the samples were placed in the center of Petri dishes. *E. coli* and *E. faecalis* dishes were incubated at 44 °C for 24 h and 36 °C for 48 h, respectively. The diameter of inhibition halos (IDs) in relation to Petri dish diameter (DD) (6 cm) was measured (Figure S2). Four measures for each sample were carried out in to determine the mean Standard Deviation. Results are expressed as Bacterial Viability (in percentage) = [(DD-IDs)/(DD)] × 100. Bacterial viability in the control plate, i.e., without geopolymeric samples, is expressed as 100%. The mean Standard Deviation is expressed as Relative Standard Deviation (RSD).

#### 2.3.8. Cytotoxicity Assessment

The colorimetric tetrazolium dye MTT (3-(4,5-dimethylthiazol-2-yl)-2,5-diphenyltetrazolium bromide) assay was used to determine the redox mitochondrial activity on NIH-3T3 murine fibroblast cells. To this purpose, the cells were grown in Dulbecco’s Modified Eagle Medium supplemented with 10% fetal bovine serum, 50.0 U/mL penicillin, and 100.0 μg/mL streptomycin, at 37 °C in a humidified atmosphere containing 5% CO_2_. The cells were seeded at a density equal to 3.0 × 10^5^ per well, onto 6-well plates, and then, they were directly exposed to the synthesized materials (1.0 mg). After 2, 6, and 48 h of incubation, cells were treated with MTT (500 μL; 0.50 mg/mL) dissolved in the culture medium. MTT solution allowed to be in contact for 2 h at 37 °C in the 5% CO_2_ humidified atmosphere. Then MTT solution was detached, and DMSO was used to dissolve formazan. The absorbance at 570 nm of each well was measured by a Victor3 Perkin Elmer fluorescence and absorbance reader (Waltham, MA, USA). The cell viability was articulated as a percentage of mitochondrial redox activity of the cells directly visible to powders, compared to an untreated control.

## 3. Results

### 3.1. Sample Observation

The molds containing the GP and GP/WG (20, 50 and 60 wt%) with fine and coarse glass grain size ranges were opened and extracted at room temperature after 7, 14, and 28 curing days. (Images of the specimens are reported in the Supplementary Materials as Figures S3 and S4, showing the sample inside the mold and after the extraction).

Visual inspection of all the samples after the extraction from the mold at different curing times is reported in the following. Once extracted, GP and GP/WG (20%) samples appeared very wet, hard, smooth, and finger pressure resistant. The GP/WG (50 and 60%) samples were less wet and hard. Moreover, the GP/WG 60% (both grain sizes) and GP/WG 50%-coarse size samples broke during the extraction procedures, and the samples showed probably un-reacted sodium silicate on the upper base (as is possible to see in the supplementary section Figures S3D,H,L and S4C,D,G,H,K,L). Comparing both grain sizes, all the samples passed the integrity test (see Figures S5 and S6) with exception of the formulations: GP/WG 50% and 60% (37 µm < d_WG_ < 53 µm and 75 µm < d_WG_ < 105 µm), that were not finger pressure resistant after the test at 7 and 14 days of curing times, while they needed at least 28 days to stabilize their structure.

**Figure 1:**
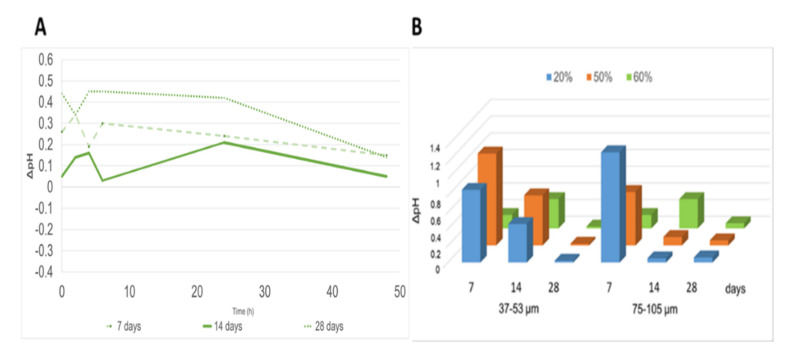
∆pH with GP reported: (**A**) vs. time for GP/WG 60% (37–53 µm) aged 7, 14, and 28 days and (**B**) for both series as a function of curing time and WG percentage.

**Figure 2:**
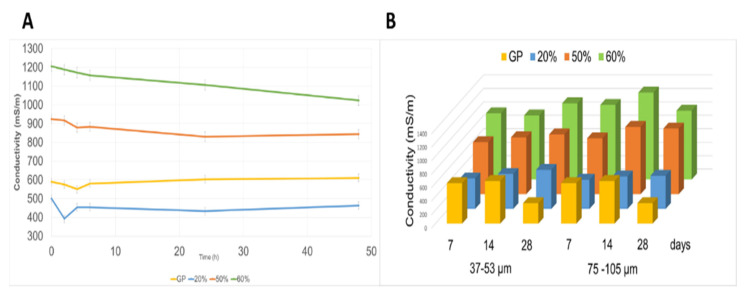
Ionic conductivity values reported: (**A**) vs. time for GP-WG (75–105 µm) aged 7 days at different WG % and (**B**) for both series as a function of curing time and WG percentage.

The values of pH (Figure 1) and ionic conductivity (Figure 2) over a period of 48 h in stirring water and with a solid/liquid ratio 1:10 were performed in order to evaluate ion release from part of all the geopolymers, as shows as an example the trend of the pH, calculated as the pH of GP/WG 60% subtracted by the pH value of GP, after 7, 14, and 28 days. Note that at 48h the difference between GP/WG and GP is about 0, thus confirming a stabilization of the pH of the WG added MK-based geopolymers. pH trend shows an initial jump, as observed in previous work [39]. All the tested formulations present similar behaviors. In Figure 1B it the pH trend between the two different grain size is very similar, confirming that after 28 days the pH of the GP/WG at various %, and particle sizes are almost identical to that of the GP based only on MK. The finer WG reacts faster, showing a ΔpH close to zero after a curing time positioned between 14 and 28 days.

The trend of the ionic conductivity increases as the % of glass increases (Figure 2A) and remains constant over time except in the GP after 28 days (Figure 2B). Furthermore, the trend of the samples with the two different WG grain sizes is similar.

**Figure 3:**
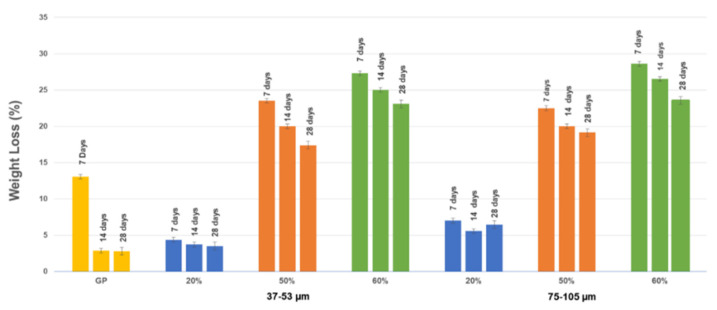
Values of weight loss after the integrity tests for samples GP and GP/WG from 37–53 µm and 75–105 µm series.

The weight loss tests were carried out on each sample three times, i.e., with three different pieces of the same sample, thus managing to obtain an acceptable error. Representative images of samples after weight loss test are reported in supplementary section as Figures S7 and S8. All the samples show a very good stability, starting from 7 days. Figure 3 shows the values of weight loss after the integrity tests for samples GP and GP/WG for each grain size range. The trend is almost similar for the GP/WG in both series moving from 20% to 50 and 60%. The increase in weight loss with the increase in the % of waste glass coincides with the properties observed at extraction from the mold: GP/WG 60% showed more cracks than the GP/WG 20% indicating a visibly lower degree of consolidation.

### 3.2. FT-IR Microstructural Characterization

One of the characterization techniques most sensitive to the features of aluminosilicate chemical bonds is Fourier Transform Infra-Red spectroscopy (FT-IR). Several studies have been already published for the interpretation of metakaolin based geopolymers (Table 1) [8].

**Figure 4:**
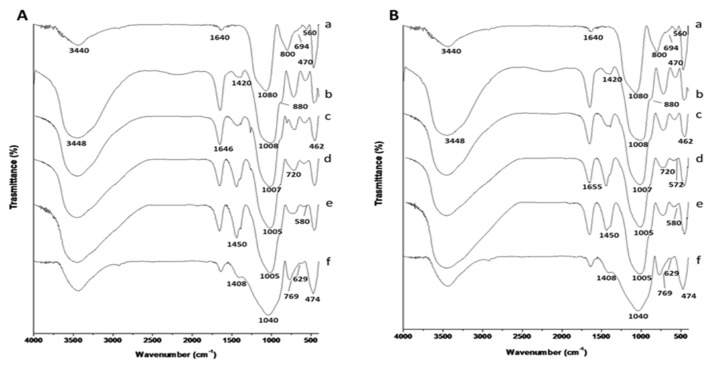
FT-IR spectra of (a) MK, (b) GP, (c) GP/WG 20%, (d) GP/WG 50%, (e) GP/WG 60%, and (f) WG at 28 days of curing time for (**A**) 37–53 µm and (**B**) 75–105 µm grain size.

**Figure 5:**
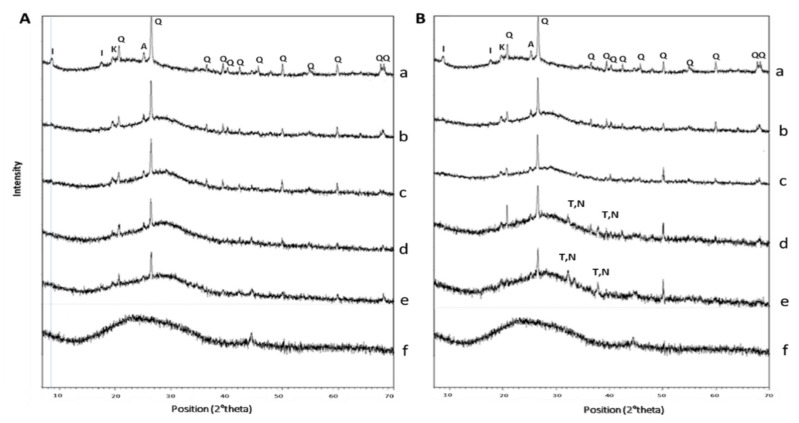
XRD patterns for samples GP, WG, and GP/WG from series: (**A**) 37–53 µm and (**B**) 75–105 µm. Pattern a = MK; b = GP; c = GP/WG20%; d = GP/WG50%; e = GP/WG60% f = WG. Crystalline phases identification label: Q = Quartz, K = Kaolinite, I = Illite, A = Anatase, T = Thermonatrite and N = Trona Na_3_(HCO_3_) (CO_3_)·2H_2_O.

**Figure 6:**
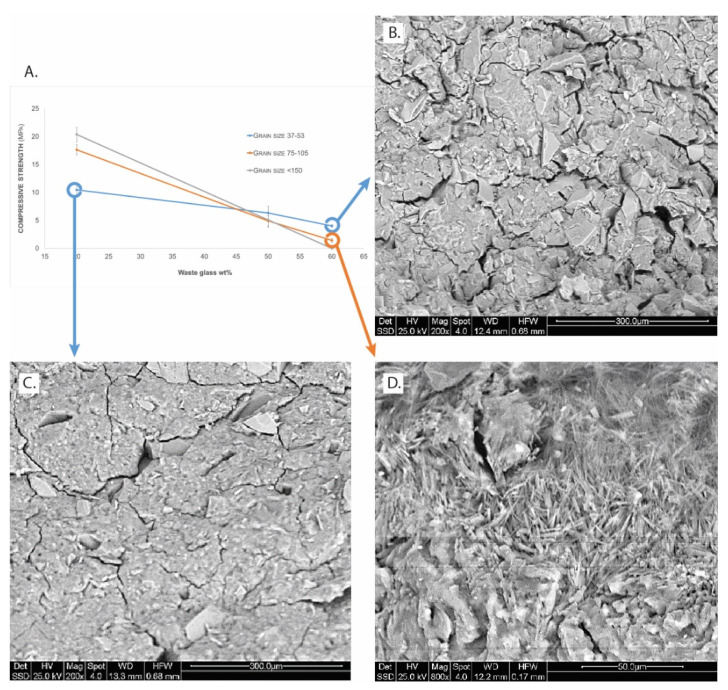
(**A**) Compressive strength for all the samples aged 28 days (lines are drawn for eye help only). SEM observation of fresh fractured surfaces from samples with finer grain size: 37–53 µm (**B**) GP/WG 60%, (**C**) GP/WG 20% (BSE, 800×), (**D**) GP/WG 60% with coarse gain size (BSE 800×).

Figure 4 shows FT-IR spectra of MK, WG, GP, and GP/WG (20, 50, and 60%) at 28 days of curing time, for the two different WG grain sizes. In MK spectrum (Figure 4A(a) or Figure 4B(a), both spectra are referred to as-received MK), the bands at 344 cm^−1^ and 1640 cm^−1^ are assigned to –OH stretching and bending vibration of water’s hydration [40,41]. The peak at 1080 cm^−1^ is assigned to Si–O–Si or Si–O–Al asymmetric stretching vibrations [8]. The Si–O bands observed at 800 and 694 cm^−1^ and 470 cm^−1^ indicate the presence of quartz [41] (see also XRD patterns in Figure 5). The absorption band at 560 cm^−1^ could be related to the existence of Al–O vibrations of Al in six-fold coordination [41,42], due to the presence of illite in MK (see also XRD patterns in Figure 6) [41]. FT-IR spectra of GP and GP/WG (20, 50 and 60%) samples (both WG grain sizes) are exposed in Figure 4A,B(b–e). In all of spectra, the carbonate group presence (1450–1420 cm^−1^), which is absent in MK, is detectable [41,43] (as reported in Table S1 in Supplementary Materials).

The GP and GP/WG peak at 880 cm^−1^ are assigned to both Si–OH bending [43] and Al(V)–O stretching [40], while the band around 720 cm^−1^ is assigned to Si–O–Al bending vibration [41]. The presence of Si–O–Al framework is also confirmed by the presence of the bands at 580–570 cm^−1^ [43]. Finally, the bands at 470–450 cm^−1^ are assigned to Si–O–Si and O–Si–O bending [40,41].

### 3.3. Mineralogical Composition

The diffraction pattern from metakaolin (Figure 5) shows the typical diffuse reflection of the amorphous aluminosilicate structure plus shaper peaks identified as anatase, and alpha-quartz, present in the sample. The XRD patterns from the samples GP/WG50 and GP/WG60 with coarse glass size series were found to have one or two additional reflections, indicating that one or more crystalline phases formed as consequence of alkali activation.

In details, the mineralogical composition of MK indicates the presence of α-quartz SiO_2_ (PDF 089–1961) with very small traces of anatase TiO_2_ (PDF 075–2547), and illite (K,H_3_O)(Al,Mg,Fe)_2_(Si,Al)_4_O_10_[(OH)_2_,(H_2_O)] (PDF 043–0685) (Figure 5, diffraction pattern indicated as f).

The diffraction patterns from GP and GP/WG series were similar, and both had a diffuse reflections characteristic of amorphous geopolymer at about 26–28° in 2θ [44] (Figure 5A,B). Metakaolin also had a diffuse reflection at lower 2θ, 17–22°, which transformed into the geopolymer amorphous halo following the polycondensation reaction. The amorphous nature of GP and GP/WG series was in agreement with results published by Fletcher et al. 2005 [45]. The shift towards high 2θ values of the amorphous halo is more pronounced for the fine WG, indicating a more reactive glassy powders into the formulation.

XRD patterns exhibited carbonate peaks around 33° in 2θ (thermonatrite (Na_2_CO_3_·H_2_O) and trona Na_3_(HCO_3_)(CO_3_)·2H_2_O), in the cases of GP/WG50% and GP/WG60% with coarse powders (Figure 5B), indicating the presence of unreacted NaOH solution coming into contact with atmospheric CO_2_.

### 3.4. Mechanical Properties and Microstructure

In order to evaluate the mechanical properties of geopolymers based on metakaolin and waste glass, compressive strength measurements were carried out.

From the plot reported in Figure 6A, it can be observed that the maximum compressive load increases with increasing WG particle size only in the case of 20% additions, whereas it decreases in the cases of 50 and 60% additions. Similar results were found in a published study after 28 days aging with WG was added to fly-ash [46]. In this plot, we also reported the data from another study where a WG with size <150 mm was added to this MK. The trend is repeated: with low additions of WG powder to the GP, we observe an increase in strength, indicating a beneficial effect of glass grains in the geopolymer structure. This type of material is a composite, where the glass particles act as reinforcement, as presented in Figure 6B,C.

Additionally, SEM micrographs of the geopolymer (Figure 6B,C) clearly show that the microstructures of the samples are different: while GP/WG 60% consisted of fractured matrix, the microstructure of GP/WG 20% was much denser and more typical of a metakaolin geopolymer [47]. The absence of unreacted platy metakaolin particles and the presence of acicular Na carbonate crystals is reported in Figure 6D.

### 3.5. Antibacterial Activity

**Figure 7:**
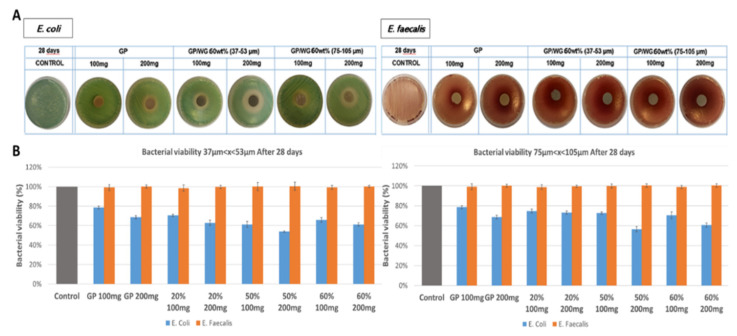
A representative image of bacterial plates (**A**) of E. coli and E. faecalis in presence of GP and GP/WG 50 wt% after 28 days curing time for fine and coarse series. (**B**) shows bacterial viability for both series.

In order to provide preliminary functional information of GP and GP/WG geopolymers, the latter were tested for their antibacterial efficacy at 7, 14 and 28 days curing time using two different strains of bacteria: a Gram-negative and a Gram-positive. The incubation temperatures, 44 and 36 °C for *E. coli* and *E. faecalis*, respectively, were chosen to allow the faster growth of the two different bacteria, differing from the experiments run by Mejía-de Gutiérrez et al. 2020 [48] when evaluating surface properties at room temperature. Figure 7 shows representative data achieved for GP and GP/WG 60% after 28 days curing time for 37–53 and 75–105 µm grain sizes. In Figure 7A it is observed an increase of *E. coli* inhibition halo on plates for all samples tested. In particular, the addition of waste glass to geopolymer appears to increase the inhibitory effect. On the contrary, all the samples showed no antibacterial properties for *E. faecalis*.

Figure 7B shows bacterial viability in presence of GP and GP/WG materials for 37–53 and 75–105 µm series. A significative *E. coli* viability reduction is observed when the quantities of samples were increased from 100 to 200 mg underlining an amount-dependence of this effect. On the contrary, *E. faecalis* viability is not affected by the presence of GP and GP/WG materials.

### 3.6. Cytotoxic Effects of GP/WG Samples

**Figure 8:**
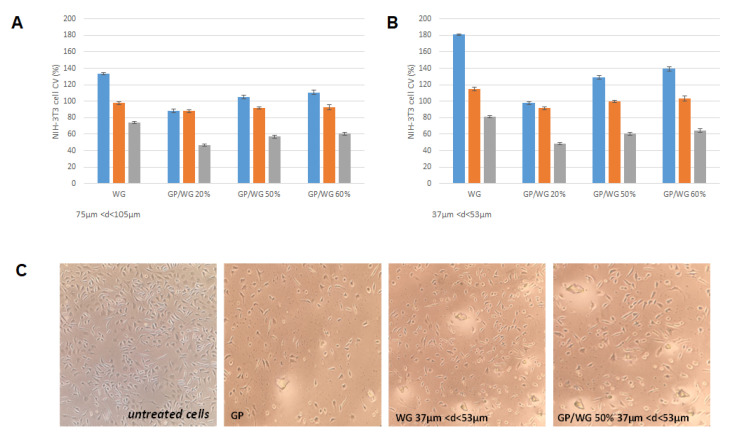
NIH-3T3 cell viability (CV, %) assessed by means of MTT assay at 2 (blue), 6 (red), and 48 (grey) hours of exposure time. (**A**) Data acquired by treating cells with 75–105 µm and GP/WG 37–53 µm (values are the mean ± SD of three independent experiments); (**B**) data acquired by treating cells with WG 37–53 µm and GP/WG 37–53 µm (values are the mean ± SD of three independent experiments). (**C**) Representative images of NIH-3T3 cells grown in absence (untreated cells) or in presence of GP, WG, 37–53 µm, and GP/WG 50%. 37–53 µm. The images were acquired after 6 h exposure time.

Cytotoxicity assessment was carried out at different exposure times (2, 6, and 48 h) in order to simulate short and long contact of NIH-3T3 cells with the synthesized materials (Figure 8). Data acquired were in accordance with a positive effect of WG incorporation in GP-based material. In particular, it was observed that an increase in WG percentage was consistent in an augmentation of redox mitochondrial activity of tested cells and that WG material’s diameter affected the cell response. The exposure time appeared to influence the cytotoxic behavior, and long exposure markedly decreased cell viability. This could be due to glass nature of the material and its reduction as powders for performing MTT assay. Glass material could break cell integrity affording the negative outcome, especially when the cells are exposed for a much longer time. GP/WG treatment appeared to compromise the cell morphology. Representatively, the cells treated with GP/WG materials for 6 hours’ exposure time are reported in Figure 8C. The cells displayed a more or less spindle-like morphology.

## 4. Discussion

The formulations tested in this study have showed a good chemical stability not showing any efflorescence after curing and good chemical stability at waste glass content of about 20wt% (Figure 1, Figure 2 and Figure 3, and Supplementary Materials Figures S3, S4 and S6). Efflorescence is still absent for geopolymers with WG content higher than 20wt%, for both grain size ranges, while chemical stability is reduced as well as mechanical performance (Figure 6A). The overall densification is, nevertheless, very relevant also for samples containing up to 60 wt% of waste glass from food packaging, indicating the possibility to use these formulations as non-structural materials for indoor applications.

**Figure 9:**
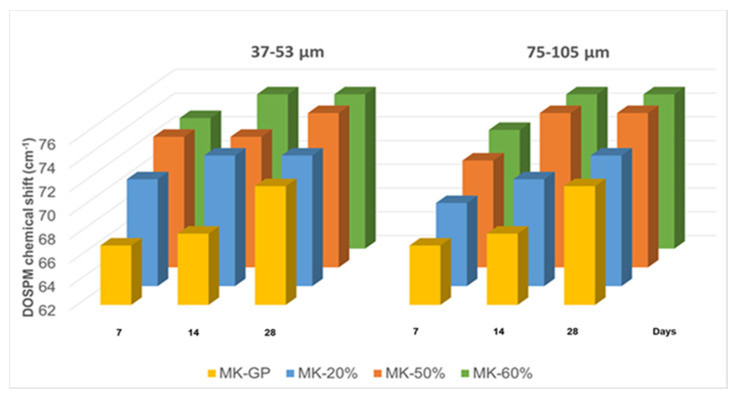
DOSPM chemical shift from MK of all the samples as function of curing time and WG size.

Particular attention has been posed on the role of the WG in its finer grain size range, 35 μm < d_WG_ < 57 μm, where the interaction with the alkaline solution is more intimate with respect to coarse grains. The reactivity of fine WG powders within the geopolymer gel has been carefully examined and proved via FT-IR (Figure 4) specially with the bands between 1008 and 1005 cm^−1^ ascribed to Si–O–Si and Si–O–Al asymmetric stretching vibrations. These bands, also known as DOSPM (Density of States Peak Maximum), are often used to determine the geopolymerization degree [49]. The DOSPM shift of GP and GP/WG (20, 50 and 60%) in respect to MK DOSPM (that appears at 1080 cm^−1^) is reported in Figure 9 and gives an indication of MK dissolution and polycondensation reaction which lead to the formation of specific geopolymer network [41]. Moreover, the lack of DOSPM sharp features (Figure 9) is also indicative of the general disorder in the Si(Al)-O-network, reflecting the wide distribution of SiQn(mAl) units either in the geopolymer or in WG/MK. SiQn(mAl) is used to describe the structural units in aluminosilicates, in which, generally, the absorption bands of the SiQn unit with n = 4,3,2,1,0 are centered at around 1200, 1100, 950, 900, and 850 cm^−1^, respectively. The shifts to lower wavenumbers occurred when the degree of silicon substitution by aluminum in the second coordination sphere increases, as consequence of the weaker Al–O bond [49].

FT-IR spectra (1400–750 cm^−1^) of MK and GP/WG (20, 50 and 60%), both grain sizes at 28 days of curing time, were deconvoluted (Figure S9). Deconvolution was done to better realize the effect of increasing amount of WG on geopolymerization process, compared to metakaolin. Fitting was done using Gaussian equations with a regression coefficient (R^2^) from 0.9989 to 0.9999. In the deconvoluted spectrum of MK (Figure S9a,b), the band arising at 812 cm^−1^ is due to O–Al–O bending vibrations of AlO_4_ tetrahedra. The strong band at 1065 cm^−1^ is a major feature of metakaolin due to the asymmetric stretching modes of Si–O, which is also associated with two satellite bands at 1195 and 998 cm^−1^ [50] due to different structural units with the same silicate framework [51]. After geopolymerization with different WG content, bands at 876–871 cm^−1^ are due to OH bending in Si–OH groups [52]. The principal band at 1038–1029 cm^−1^ (Figure S9c–h) is due to the asymmetric stretching of Si–O–T links in geopolymers. Hajimohammadi et al. stated that this band is sensitive to connectivity and Si/Al ratio. Moreover, they revealed that this band can be attributed to the presence of predominantly Si–O–Al bonds. The new bands at 961–951 cm^−1^ (Figure S9c–h) are assigned to the asymmetric stretching vibration of non-bridging oxygen sites, in particular Si–O–Na type structure [52]. The bands at high wavenumber (1148–1140 cm^−1^), in Figure S9c–h, are an indication of more participation of silica in the later stages of gel cross linking and reorganizing [53]. Finally, bands arising at 1264–1253 cm^−1^ are consistent with T–O–Si asymmetric stretching in the un-reacted metakaolin [54]. In all GP/WG samples, deconvolution analyses confirmed similar structures regardless the WG content and grain sizes.

The degree of reactivity of the WG with the alkaline media can be also related to the amount of sodium carbonate formed by the reactivity of unreacted of NaOH and atmospheric CO_2_ [55,56]. The formation of a sodium carbonate phase on geopolymer samples can be explained by the reaction of unreacted NaOH that interacts with CO_2_ present in the atmosphere after the extraction of the sample from the mold. Shim et al. [57] explored the possibility to produceNa_2_CO_3_ by the reaction starting from NaOH (aq) and CO_2_ (g). According to the authors, the reaction is 2NaOH(aq)+ CO_2_(g) -> Na_2_CO_3_ (aq) + H_2_O(l) and has a ∆G=-128.97 kJ/mol. The occurrence of sodium carbonate formation increases with the increase of the waste glass content and its grain size as a result of the increment of unreacted NaOH during the geopolymerization. The design of the geopolymer formulations considered the waste glass as a substitute for metakaolin leaving the alkali solution fixed to a constant value (48 mL of NaOH plus 68 mL of sodium silicate). The amount of the waste glass and its grain size led to different reactivity with respect to metakaolin, so the same NaOH quantity acts differently in the geopolymer formulations used (see also [19]). The occurrence of sodium carbonate is also in accordance with XRD spectra of the samples obtained with high percentages of waste glass showing thermonatrite and trona peaks. These peaks are not present in the starting waste glass and in the geopolymers with low waste percentages and low grain size. Moreover, in these systems, the Na-ions are believed to be related to the Si–O–Al framework of the geopolymeric gel, contributing to balance the negative charge associated with tetrahedral Al(III) [41]. The excess of Na^+^ eventually reacts with CO_2_ to originate the carbonatic phase. Such salt is absent in a relevant amount, being absent any trace of efflorescence. The presence of sodium carbonates has been investigated by XRD, where thermonatrite (Na_2_CO_3_·H_2_O) and trona Na_3_(HCO_3_)(CO_3_)·2H_2_O) have been found in the cases of GP/WG50% and GP/WG60% with coarse powders (Figure 5B). Additionally, in the XRD diffraction, no other novel crystalline phases have been recognized. Thus we assessed that the presence of WG did not alter the amorphousness of the geopolymeric gel, as stated by FT-IR deconvolution. The shift in the position in 2 theta of the amorphous halo can be attributed to the presence of high amount Al-O–Si bonds in the geopolymer gel with respect to the Si–O–Si, typical of the container soda-lime-glass [45]. This experimental result supports the evidences in the 1080–1005 cm^−1^ peak, as already commented.

With SEM observations, we observed the different microstructures’ impact on physico-chemical differences of the samples such as compressive strength and water leachability. When the fraction of WG powders substituting the MK in the geopolymer matrix is too high, this reinforcement role is lacking. The coarse WG grains are not reactive enough to use the alkaline solution added to the formulation, as indicated by the presence of the Na carbonate crystals (Figure 6D). For all the formulations, the absence of unreacted MK plate-like grains was checked at high enlargements (Figure S10A).

Wanting to deepen the chemical nature of the geopolymer matrix, we proceeded to elemental analysis via EDS (Figure S10B). The geopolymer gel/matrix in sample GP/WG 20% (Figure 6C) contains: 18 Na_2_O, 24 Al_2_O_3_ and 58 SiO_2_ (wt%), while in sample GP/WG 60% (Figure 6B) the EDS semi-quantitative analysis gave the values of 22 Na_2_O, 16 Al_2_O_3_, and 62 SiO_2_ (wt%). From these values, the Si:Al molar ratio was evaluated to be equal to 2.0 and Na:Al molar ratio equal to 1.2 for formulation GP/WG 20%. In the case of GP/WG 60%, the values of the molar ratio are Si:Al=3.2 and Na:Al=2.2.

According to the classification scheme of geopolymer network proposed by Davidovits [58], when Si:Al = 1, it is a sialate (–Si–O–Al–O–); for Si:Al = 2, it is a sialate-siloxo (–Si–O–Si–Al–O); for Si:Al = 3, it is a sialate-disiloxo (–Si–O–Al–O–Si–O–Si–), and when Si:Al > 3, it is deemed to be sialate link, poly(sialate-multisiloxo). The experimental Si:Al = 2.0 for GP/WG 20% sample described in this paper indicates a good alternation of Si and Al, as shown in FT-IR Figure S9c,d while for sample GP/WG 60%, the value is a little higher than Si:Al = 3, as shown in FT-IR in Figure S9g,h. Concerning sample GP/WG 50%, the Si:Al value lies in between the sialate-siloxo and sialate-disiloxo units. However, not all the waste glass precursor is available to participate in the geopolymerisation reaction due to incomplete dissolution (glass debris are still present in Figure 6B,C). Therefore, it can be assumed that the coarser particle sizes show even a more reduced reactivity of the WG particles. In other words, we expect a Si:Al value even higher than 3.

Finally, concerning the effect of waste glass grain size on antibacterial test, GP and GP/WG with 75–105 µm glassy powder show the same behavior of finer WG grain size, but the latter seems to reduce mainly *E. coli* growth that reaches lower values of bacterial viability. Considering that antibiotic resistance in *E. coli* is of particular concern [59], the availability of materials with dose-increasing antimicrobial effect is promising to construct new sustainable antimicrobial packaging systems. Therefore, these preliminary data open up to increase the mechanism that is liable to antimicrobial response.

## 5. Conclusions

Alkali activation was proven to be a straightforward method for processing wasteglass, with grain size at least minor than 105 μm, into homogeneous dense ceramic-like geopolymers. Based on data obtained from microstructural, mechanical, and biological analyses on geopolymer containing different percentages and with different grain size of waste glass, we can conclude that the incorporation of up to 50wt% of waste container glass in MK-based geopolymer represents an excellent strategy to reduce its environmental impact. Waste glass powder acts as a filler without altering the geopolymerization process, as it is shown from FT-IR results. No substantial difference is observed using a waste glass fine (37 µm < diameter < 53 µm) grain for geopolymer synthesis, except that the finer the glass, the more reactive it appears in the alkaline environment with denser matrices of the binder, as shown by SEM observations. The preliminary antibacterial screening highlights that GP/WG are able to inhibit in a dose-increasing manner *E. coli* Gram-negative bacteria growth, while they appear ineffective with *E. faecalis* Gram-positive bacterium. The material evaluated possess alkali pH values when in contact with aqueous media. This could suggest that the geopolymer materials could affect negatively the bacterial growth as a consequence of the pH change. Moreover, Gbureck et al. [60] associated the inhibition by the materials with the high content of alkali, which increases the pH of the medium. In alkaline cements with phosphates, the inhibition reported has been 4–5 mm after 1 day of immersion in agar. Our results are in line with these observations. Herzlieb et al. [61] revealed that *Enterococcus faecalis* is not affected by alkaline condition, which is also in accordance with our data.

Moreover, the antibacterial activity can be explained by the release of ions from the surface of the geopolymer to the outer surface of the plasma membrane of the bacteria. These ions would then induce the death of bacteria interfering with the normal cell homeostasis.

The study of released ions, their quantity released from the surfaces of the geopolymer during the time, will be better investigated in new papers using theoretical methods based on Molecular Mechanics and Molecular Dynamics, which can describe at atomistic level the geopolymer surface.

When an initial biocompatibility test was performed by means of MTT direct contact test, it appeared to strongly depend on WG percentage in the synthesized materials and WG diameter, opening further investigation to clarify the GP/WG samples behavior in cell systems.

## Figures and Tables

**Table 1:** FT-IR interpretation table peaks.

Wavenumber (cm^−1^)	Vibration
3452–3440	–OH stretching
1650–1640	–OH bending
1450–1408	O–C–O stretching
1080–1005	Si–O–Si and Si–O–Al asymmetric stretching
960–950	Si–O–Na (non-bridging O)
880	Si–OH bending
800	Si–O vibration, presence of quartz
760–750	SiO_4_ vibrations
720	Si–O–Al framework
697–694	Si–O symmetric stretching, presence of quartz
580–570	Si–O–Si and Si–O–Al symmetric stretching
560	AlO_6_, presence of illite
472–450	O–Si–O or Si–O–Si bending

## Data Availability

The data presented in this study are available on request from the corresponding author.

## Supplementary Materials

The following are available online at https://www.mdpi.com/article/10.3390/polym13091493/s1. Figure S1: Comparison of grain size curves of pure MK, waste glass in the two grains sizes: 37–53 µm and 75–105 µm. Figure S2: Scheme showing the determination of the diameter of inhibition halos (IDs). Figure S3: GP and GP/WG (37 µm < d_WG_ < 53 µm) images inside the mould and after extraction for different curing times: a, b, c, d = 7days; e, f, g, h =14 days; i, j, k, l = 28 days. Figure S4: GP and GP/WG (75 µm <d_WG_< 105 µm) images inside the mould and after extraction for different curing times: a, b, c, d = 7 days; e, f, g, h =14 days; i, j, k, l = 28 days. Figure S5: Integrity Test of GP and GP/WG (37 µm < d_WG_ < 53 µm) after different curing times: s: a, b, c, d 7= days; e, f, g, h =14 days; i, j, k, l =28 days. Figure S6: Integrity Test of GP and GP/WG (75 µm < d_WG_ < 105 µm) after different curing times: s: a, b, c, d = 7 days; e, f, g, h = 14 days; i, j, k, l = 28 days. Figure S7: Weight loss of GP and GP/WG (37 µm < d_WG_ < 53 µm); a, b, c, d represent GP and GP/WG for 7 days aging time; e, f, g, h represent samples after 14 days aging time; i, j, k, l show samples after 28 days aging time. Figure S8: Weight loss of GP and GP/WG (75 µm < d_WG_ < 105 µm); a, b, c, d represent GP and GP/WG for 7 days aging time; e, f, g, h represent samples after 14 days aging time; i, j, k, l show samples after 28 days aging time, Table S1: FT-IR table peaks of MK, WG, GP and GP/WG (20, 50 and 60%) at different curing times. Figure S9: Deconvolution spectra of MK (a), GP (b), GP/WG 20%, GP/WG 50% and GP/WG 60% (c, e, g, respectively) for 37–53 µm and GP/WG 20%, GP/WG 50% and GP/WG 60% (d, f, h, respectively) 75–105 µm progression. All the samples were cured at 28 days. Figure S10: Presence of elements in MK-based geopolymers samples: (A) SEM image and (B) EDS spectra.
