# Supplementary material for: Efficient Addition of Waste Glass in MK-Based Geopolymers: Microstructure, Antibacterial and Cytotoxicity Investigation
Source: Polymers (Basel). 2021 May 6;13(9):1493. doi: 10.3390/polym13091493 (PMC8124374; doi:10.3390/polym13091493)
Supplement: Supplementary file 1 [file polymers-13-01493-s001.zip › polymers-1197781-supplementary.pdf]

SUPPLEMENTARY Polymers Dal Poggetto

Efficient addition of container waste glass in MK-based geo-polymers: microstructure, antibacterial and cytotoxicity investigation

Giovanni Dal Poggetto[1]*, Michelina Catauro[2], Giuseppina Crescente[3] and Cristina Leonelli[1]

[1]Department of Engineering “Enzo Ferrari”, University of Modena and Reggio Emilia, Via P. Vivarelli n. 10, 41125 Modena, Italy. giovanni.dalpoggetto@unimore.it; cristina.leonelli@unimore.it

[2]Department of Industrial and Information Engineering, University of Campania "Luigi Vanvitelli", Via Roma n. 29, 81031 Aversa, Italy. michelina.catauro@unicampania.it

[3]Department of Environmental, Biological and Pharmaceutical Sciences and Technologies, University of Campania "Luigi Vanvitelli", via Vivaldi n. 43, 81100 Caserta, Italy. giuseppina.crescente@unicampania.it

* Correspondence: giovanni.dalpoggetto@unimore.it

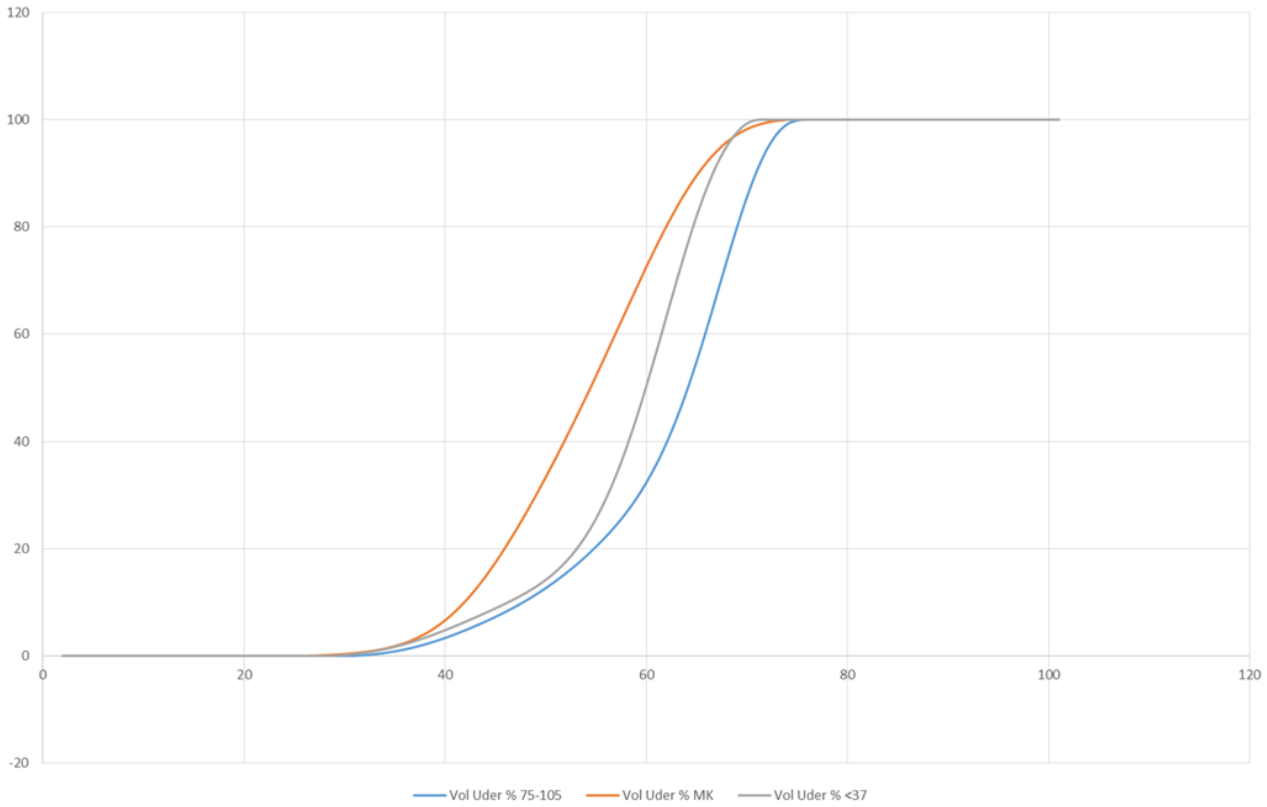

Supplementary Figure S1. Comparison of grain size curves of pure MK, waste glass in the two grains sizes: <37µm and 75-105µm.

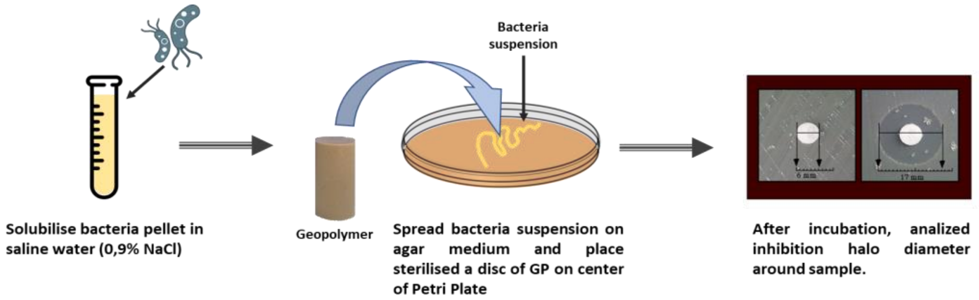

Supplementary Figure S2.Scheme showing the determination of the diameter of inhibition halos (IDs) .

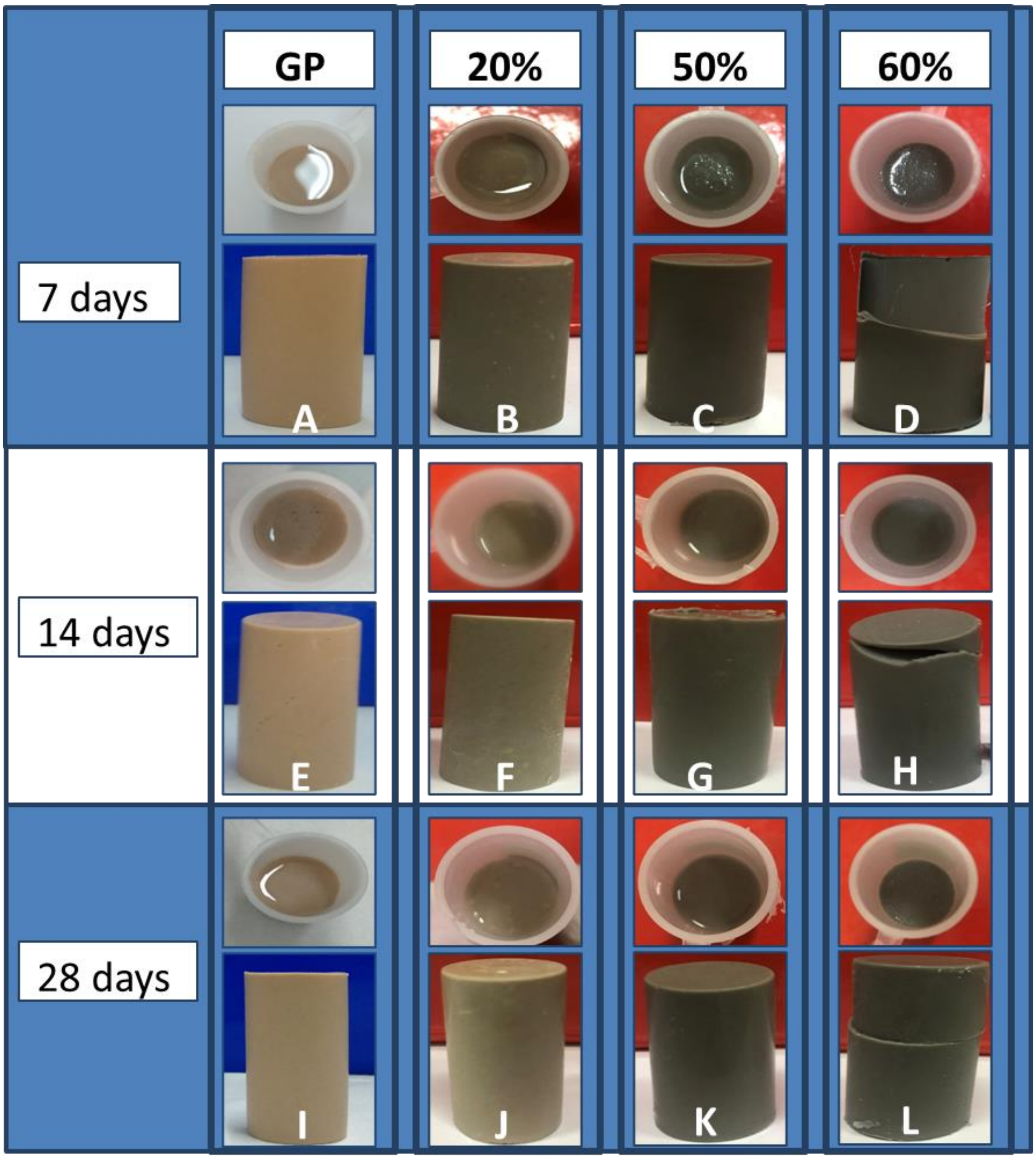

Figure S3. GP and GP/WG (37µm <d_{WG}<53µm) images inside the mould and after extraction for different curing times: a, b, c, d 7= days; e, f, g, h =14 days; i, j, k, l =28 days.

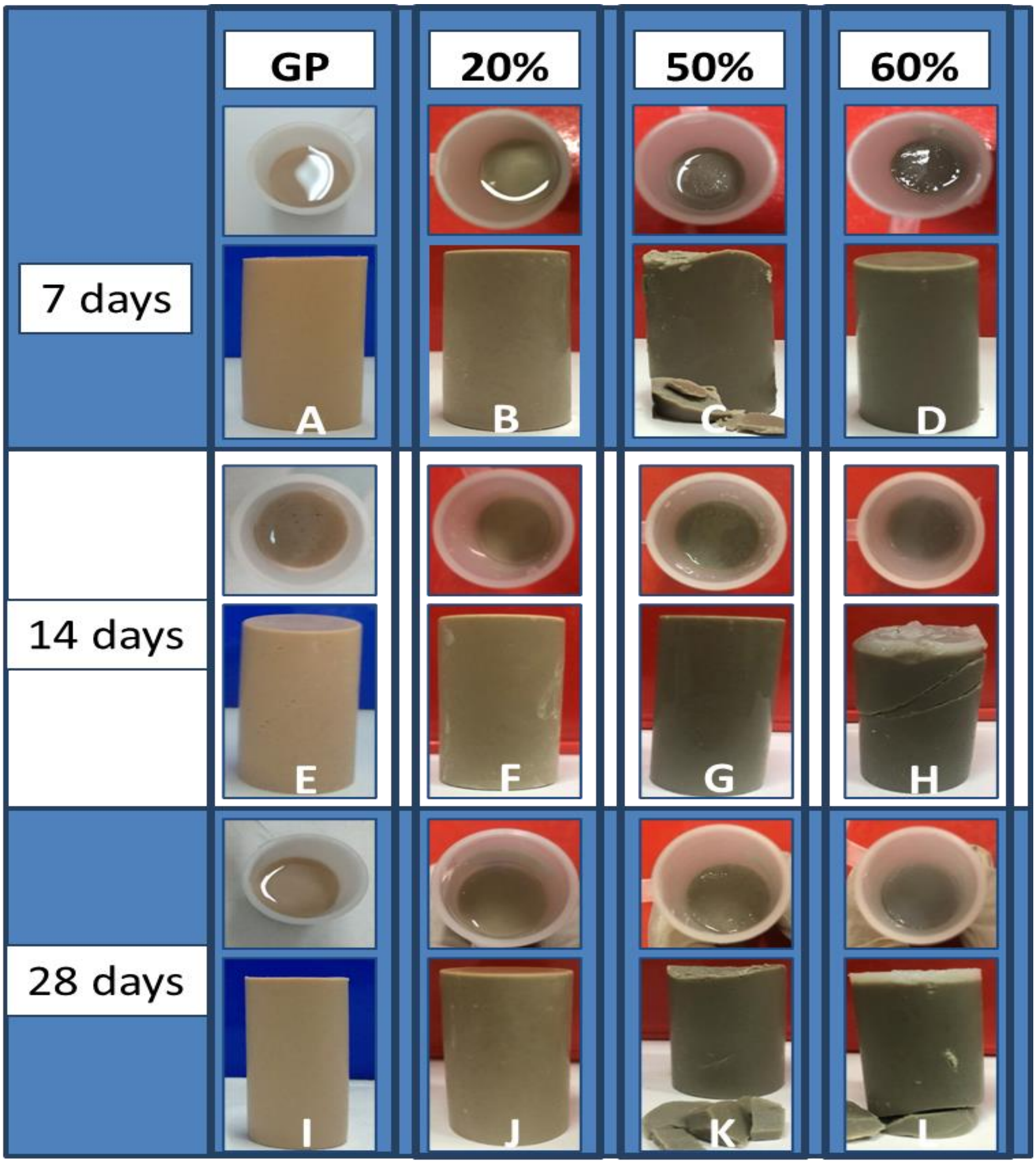

Figure S4. GP and GP/WG (75µm $<d_{WG}<$105µm) images inside the mould and after extraction for different curing times: a, b, c, d 7= days; e, f, g, h =14 days; i, j, k, l =28 days.

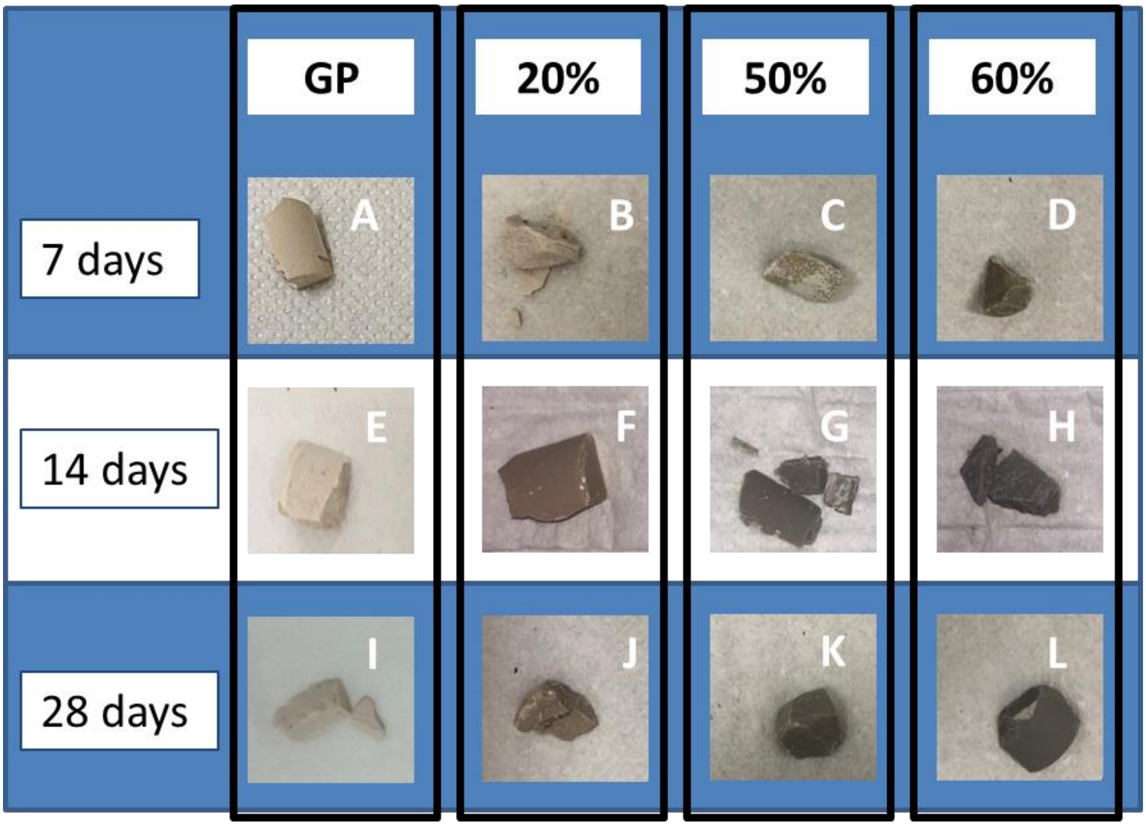

Figure S5. Integrity Test of GP and GP/WG (37µm <dWG<53µm) after different curing times: s: a, b, c, d 7= days; e, f, g, h =14 days; i, j, k, l =28 days.

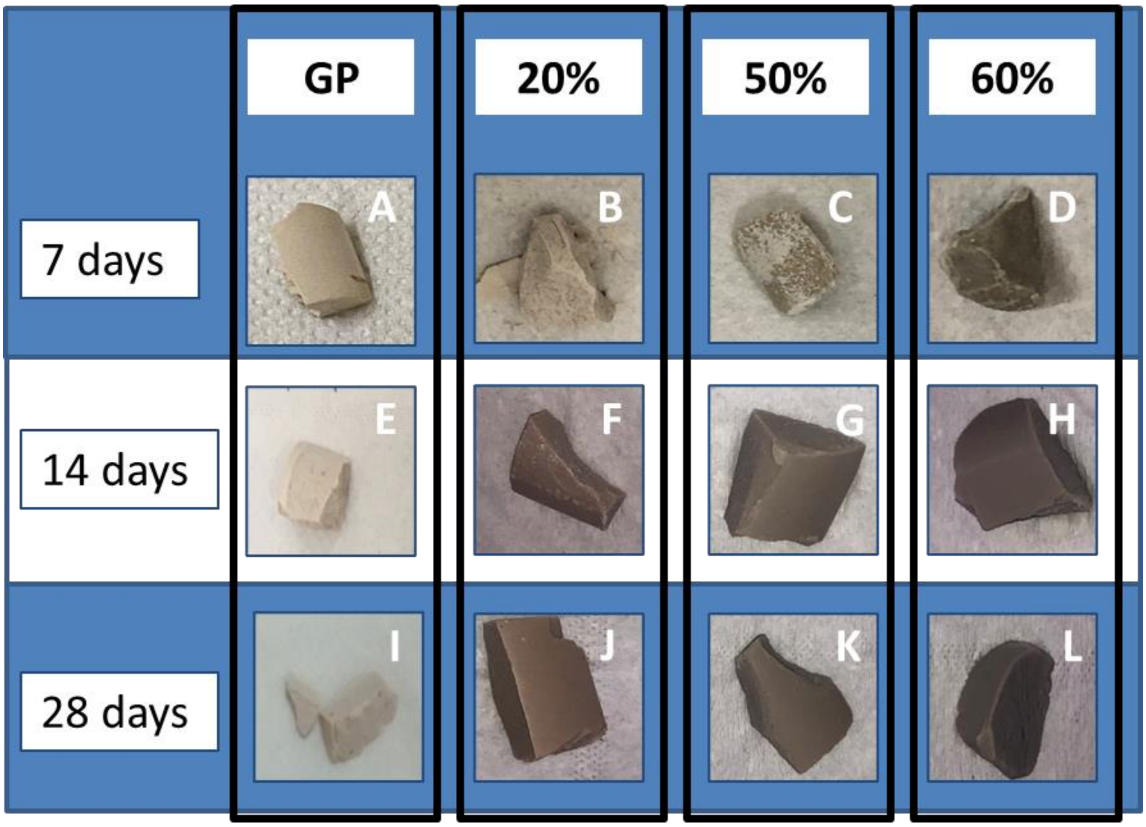

Figure S6. Integrity Test of GP and GP/WG (75µm <dWG<105µm) after different curing times: s: a, b, c, d 7= days; e, f, g, h =14 days; i, j, k, l =28 days.

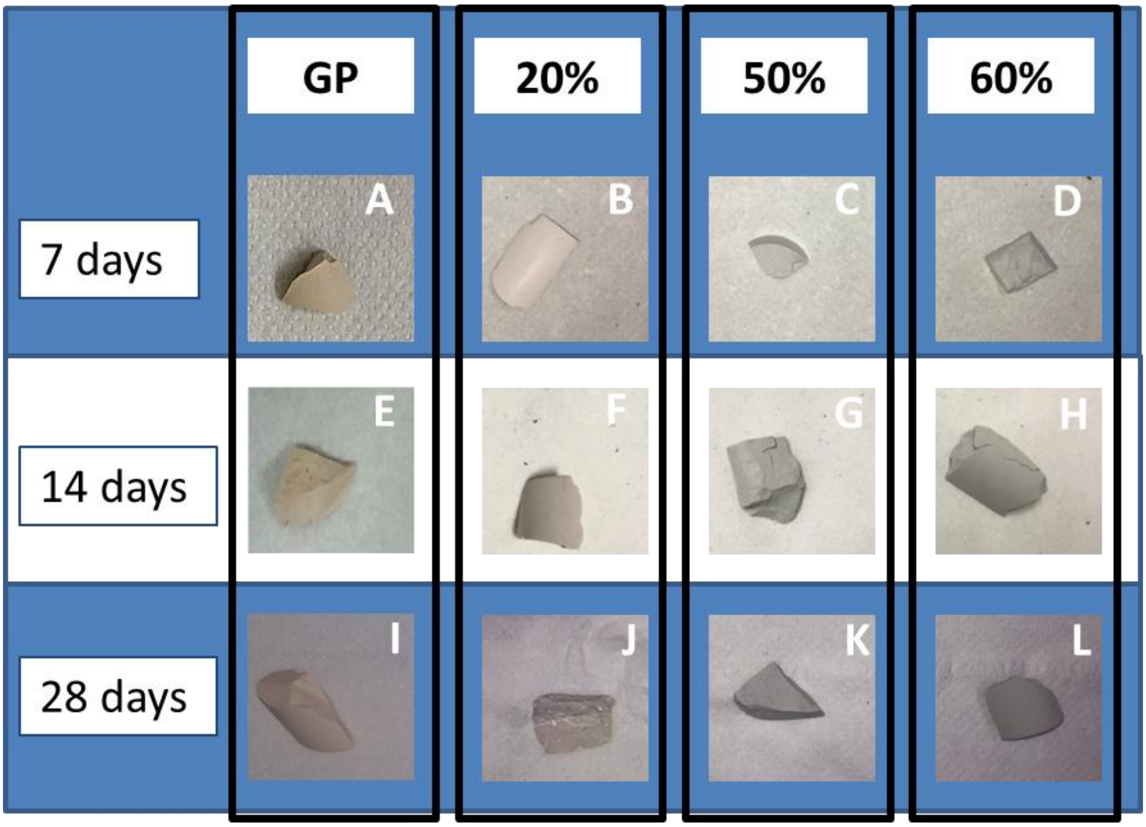

Figure S7. Weight loss of GP and GP/WG (37µm <d_{WG}<53µm); a, b, c, d represent GP and GP/WG for 7 days aging time; e, f, g, h represent samples after 14 days aging time; i, j, k, l show samples after 28 days aging time;

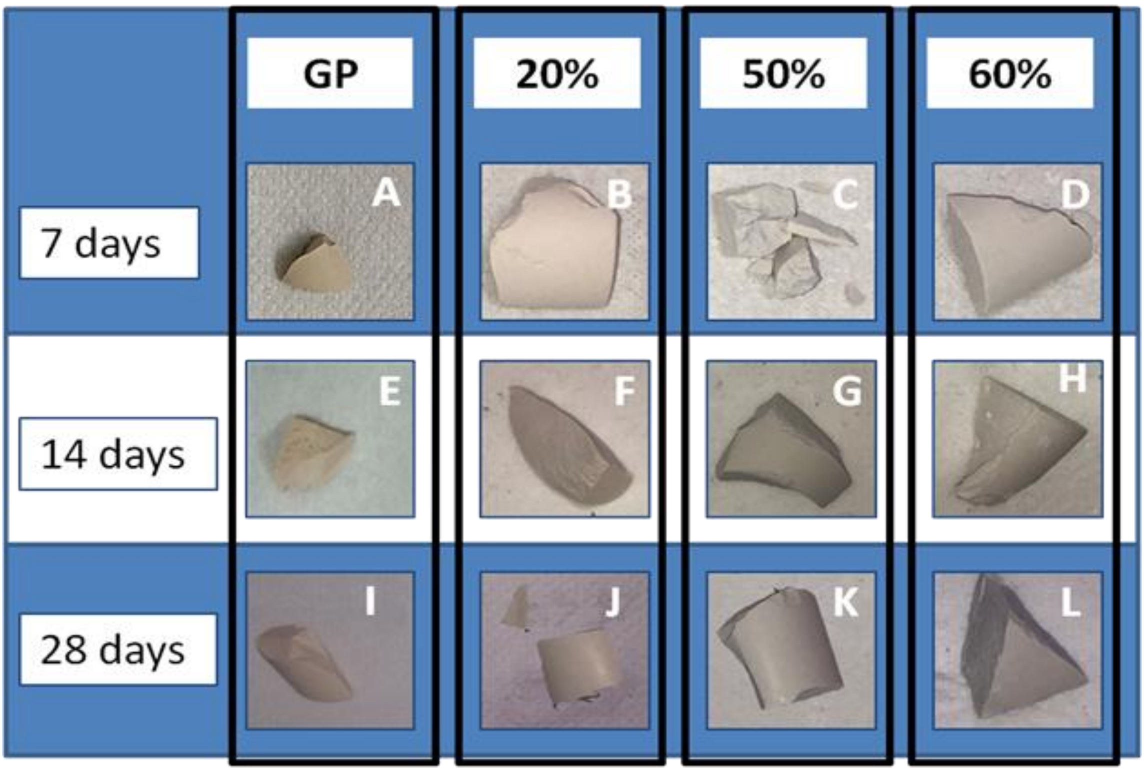

Figure S8. Weight loss of GP and GP/WG (75µm <d_{WG}<105µm); a, b, c, d represent GP and GP/WG for 7 days aging time; e, f, g, h represent samples after 14 days aging time; i, j, k, l show samples after 28 days aging time;

Aging time (days)	Sample	Peak table										
	MK	3440	1640	-	1080	-	-	800	-	694	560	470
	WG	3446	1640	1408	1040	-	-	-	769	629	-	474
7	GP	3448	1650	1450	1013	950	881	800	721	-	580	462
	20% 37-53 µm	3450	1649	1450	1009	950	880	800	721	-	571	451
	20% 75-105 µm	3449	1647	1450	1011	950	882	800	719	-	571	452
	50% 37-53 µm	3446	1647	1450	1007	950	880	800	740	696	580	451
	50% 75-105 µm	3447	1653	1450	1009	950	881	800	721	-	579	453
	60% 37-53 µm	3450	1643	1450	1007	950	882	800	750	696	580	451
	60% 75-105 µm	3451	1649	1450	1008	950	881	800	760	696		453
14	GP	3450	1651	1420	1012	950	881	800	721	-	560	462
	20% 37-53 µm	3451	1649	1450	1007	950	882	800	718	-	572	450
	20% 75-105 µm	3448	1651	1450	1009	950	882	800	712	-	571	451
	50% 37-53 µm	3447	1647	1450	1007	950	880	800	740	696	579	450
	50% 75-105 µm	3447	1650	1450	1005	950	880	800	760	-	580	451
	60% 37-53 µm	3450	1645	1450	1005	950	880	800	760	697	578	451
	60% 75-105 µm	3450	1646	1450	1005	950	880	800	760	697	580	453
28	GP	3448	1651	1420	1008	950	880	800	722	-	580	462
	20% 37-53 µm	3452	1646	1430	1007	950	882	800	712	-	580	450
	20% 75-105 µm	3449	1651	1430	1007	950	882	800	720	-	572	451
	50% 37-53 µm	3446	1647	1450	1005	950	881	800	720	-	580	452
	50% 75-105 µm	3446	1655	1450	1005	950	880	800	750	720	578	452
	60% 37-53 µm	3452	1645	1450	1005	950	880	800	760	696	581	450
	60% 75-105 µm	3451	1649	1450	1005	950	880	800	720	696	581	453

Table S1. FT-IR table peaks of MK, WG, GP and GP/WG (20, 50 and 60%) at different curing times.

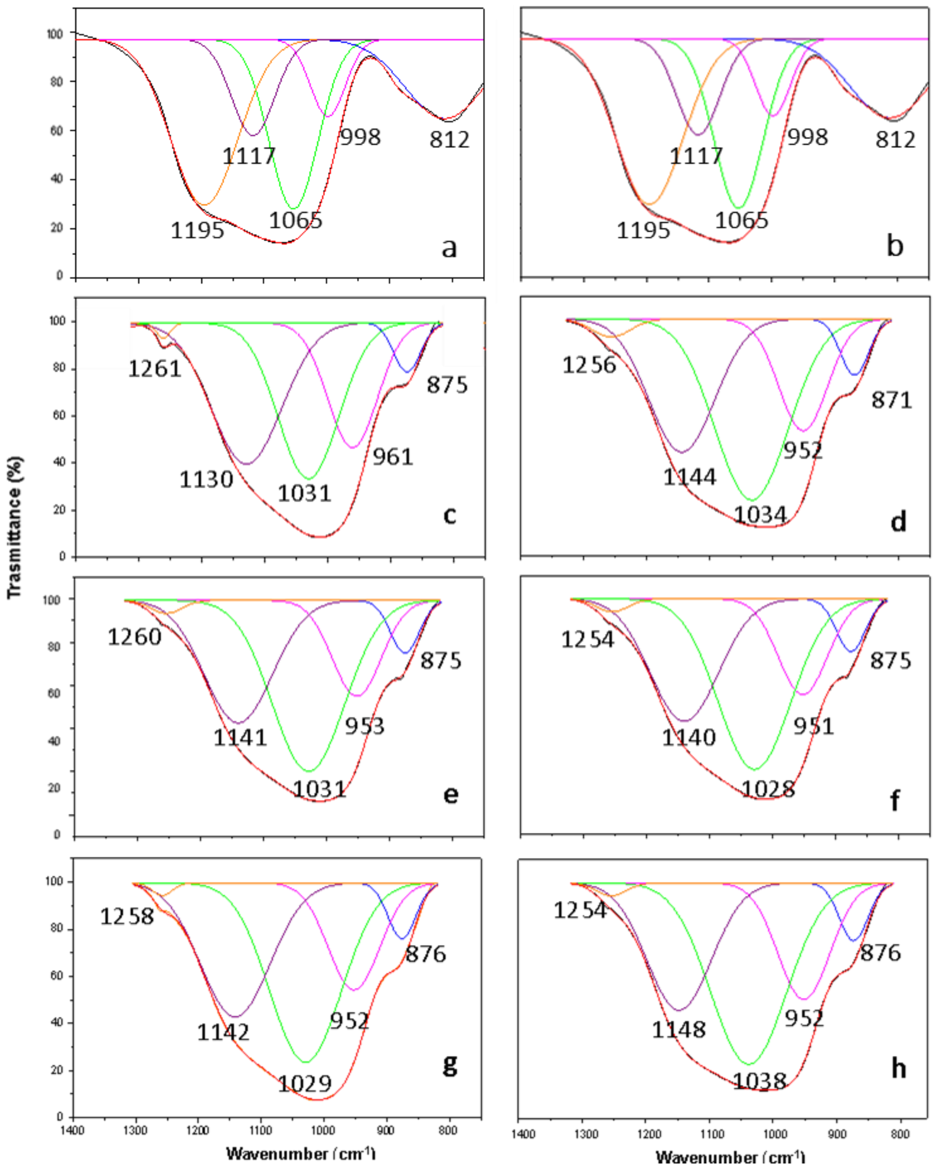

Figure S9. Deconvolution spectra of MK (a), GP (b), GP/WG 20%, GP/WG 50% and GP/WG 60% (c, e, g respectively) for 37-53 µm and GP/WG 20%, GP/WG 50% and GP/WG 60% (d, f, h respectively) 75-105 µm progression. All the samples were cured at 28 days.

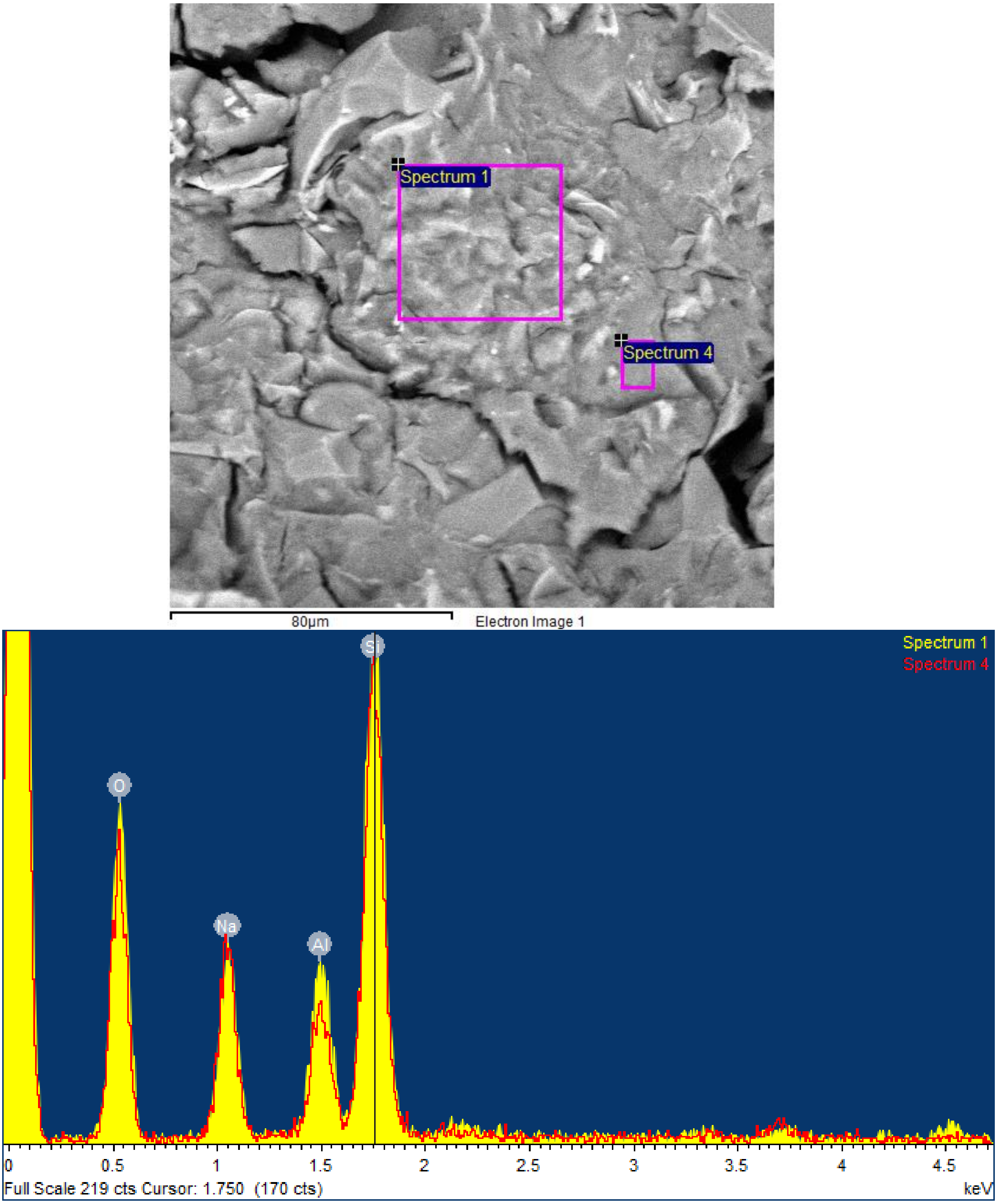

Figure S10: Presence of elements in MK-based geopolymers samples: (A) SEM image and (B) EDS spectra.
